# RNA N6-methyladenosine-modified-binding protein YTHDF1 promotes prostate cancer progression by regulating androgen function-related gene TRIM68

**DOI:** 10.1186/s40001-023-01533-5

**Published:** 2023-12-02

**Authors:** Qihong Nie, Xiaoyuan Wu, Yongming Huang, Tao Guo, Jin Kuang, Chuance Du

**Affiliations:** 1 Department of Oncology, Ganzhou People’s Hospital, Ganzhou, 341000 Jiangxi, Jiangxi China; 2 Department of Urology, Ganzhou People’s Hospital, 16 Meiguan Avenue, Ganzhou, 341000 Jiangxi China

**Keywords:** Prostate cancer, N6-methyladenosine, Androgen receptor, YTHDF1, TRIM68

## Abstract

**Purpose:**

There is no report about the direct relationship between m6A modification and androgen receptor (AR)-related genes in prostate cancer (PC). We aimed to study the mechanisms of m6A methylation in regulating the pathogenesis of PC from the perspective of AR-related genes.

**Methods:**

qRT-PCR was applied to detect the expression of m6A-related genes in PC cell with or without AR inhibitor. The effects of YTHDF1 knockdown on PC cell viability, apoptosis, migration and invasion were investigated using flow cytometry, wound healing and transwell assays, respectively. The mechanism of YTHDF1 action was investigated using m6A RNA immunoprecipitation (MeRIP) sequencing. The biological functions of YTHDF1 were also explored through in vivo experiments.

**Results:**

YTHDF1 was significantly down-regulated in AR inhibitor group. YTHDF1 knockdown significantly decreased AR level, viability and m6A methylation level of PC cells. TRIM68 was identified as a direct target of YTHDF1. Both YTHDF1 and TRIM68 knockdown increased apoptosis, and decreased cell viability, migration, and invasion of PC cells, while TRIM68 overexpression reversed the effects of YTHDF1 knockdown on PC cells. In addition, knockdown of YTHDF1 or TRIM68 significantly decreased the m6A methylation level, and mRNA and protein levels of YTHDF1, TRIM68 and AR in PC cells, while TRIM68 overexpression increased the expression levels above. Furthermore, subcutaneous xenografts of nude mice also revealed that TRIM68 could reverse the effects of YTHDF1 knockdown in PC in vivo.

**Conclusion:**

This study suggested the key role of YTHDF1-mediated m6A modification in PC progression by regulating androgen function-related gene TRIM68 in PC.

**Supplementary Information:**

The online version contains supplementary material available at 10.1186/s40001-023-01533-5.

## Highlights

1. YTHDF1 was associated with AR in PC cells.
2. YTHDF1 knockdown decreased AR level, viability and m6A methylation level of PC cells in vitro.
3. TRIM68 as a direct target of YTHDF1.
4. TRIM68 reversed the effects of YTHDF1 knockdown in PC in vitro and in vivo.

**Supplementary Information:**

The online version contains supplementary material available at 10.1186/s40001-023-01533-5.

## Introduction

Prostate cancer (PC) is a malignant tumor in genitourinary system, which is a serious threat to the male population [1]. Most patients undergo surgery, radiation, chemotherapy or hormone therapy. Despite improvements in these treatments, the 5-year recurrence rate for men with PC is still about 25 percent, and the overall mortality remains high [2]. It is well-known that the development and maintenance of the prostate depends on the action of androgen through the androgen receptor (AR) [3]. AR plays pivotal roles in PC progression [4]. About 80–90% of prostate cancers depend on androgens at the time of initial diagnosis [5]. Androgen deprivation therapy that targets AR is the primary treatment for metastatic PC and has shown therapeutic benefits for numerous patients, but patients inevitably develop into castration-resistant PC after androgen deprivation therapy [6, 7]. Exploring the molecular mechanisms associated with the role of AR in PC will accelerate the development of treatment target.

Among eukaryotes, except for the established epigenetic DNA modifications, RNA modifications are also prevalent. N6-methylladenosine (m6A) is one of the most common and reversible modifications on mRNA [8]. Now, it is well-known that m6A is regulated by the methyltransferases (writers, such as METTL14, METTL3 and WTAP), demethylases (erasers, including ALKBH5 and FTO) and RNA-binding proteins (readers, such as YTHDC1/2, YTHDF1/2/3, and IGF2BP1/2/3) [9, 10]. Dysregulation of m6A modification participates in various pathological processes, particularly in tumorigenesis, including the progression of PC. It has been reported that m6A methyltransferase METTL3 can promote the progression of PC via m6A-modified LEF1 [11]. In addition, Cai et al. [12] reported that METTL3 promoted the growth of PC. More recently, Zhu et al. [13] suggested that m6A demethylase FTO inhibited the migration and invasion of PC cells by regulating the total m6A levels. Xia et al. [14] demonstrated that m6A-induced suppression of SIAH1 facilitated alternative splicing of androgen receptor variant 7 by regulating CPSF1 in PC. To our best knowledge, there is no report about the direct relationship between m6A modification-associated genes and AR-related genes in PC. Given the important roles of AR in PC and m6A modification in PC, we speculated that m6A methylation may be associated with the pathogenesis of PC by regulating androgen function-related genes.

Thus, in this study, we first screened m6A genes that were associated with androgen function in PC cells by treating cells with AR inhibitor. Then we clarified the regulatory mechanisms of this m6A gene on the progression of PC through in vitro and in vitro experiments. Our study may provide novel directions in clinical treatment of PC.

## Results

### YTHDF1 was associated with AR and AR inhibitor inhibited proliferation of PC cells

**Fig. 1:**
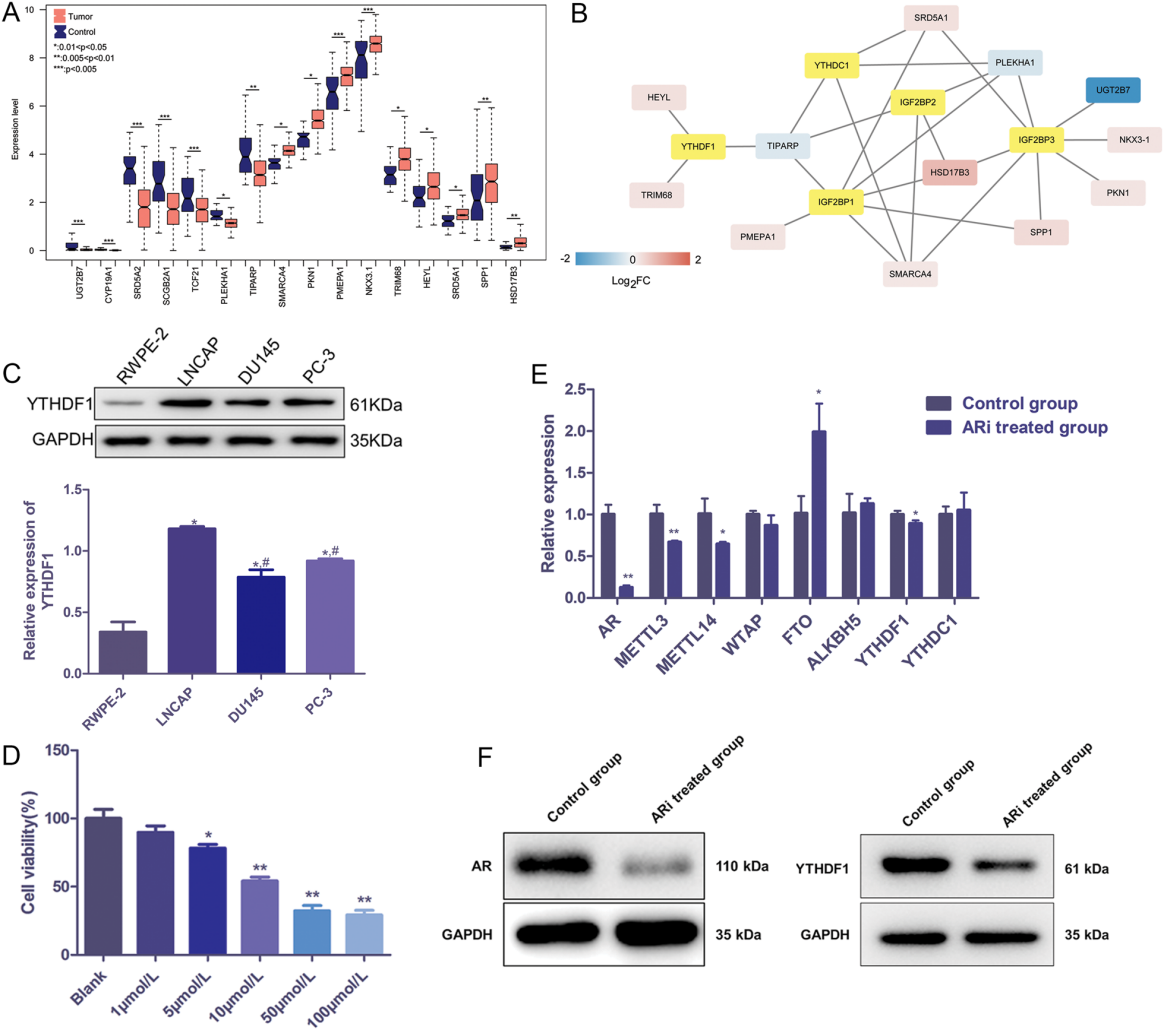
YTHDF1 was associated with AR, and androgen receptor (AR) inhibitor inhibited viability of prostatic cancer (PC) cells. **A** Significantly differentially expressed androgen-related genes. **B** Regulatory network of m6A genes and the differentially expressed androgen-related genes. Yellow nodes represent m6A genes, and the rest represent differentially expressed androgen-related genes. **C** Protein expression of YTHDF1 in the normal cells and PC cell lines detected by western blot. *P < 0.05, compared to RWPE-2; ^#^*P* < 0.05, compared to LNCAP. **D** Cell viability of LNCAP after 48 h of treatment with different concentrations of AR inhibitor. *P < 0.05, ** *P* < 0.01, compared to the blank group. **E** mRNA expression levels of m6A-related genes detected by qRT-PCR. *P < 0.05, ** *P* < 0.01, compared to the control group. **F** Protein expression levels of AR and YTHDF1 detected by western blot. ARi treated group: AR inhibitor treated group

First, bioinformatics analysis was used to select the m6A enzymes regulated genes related to androgen in PC. Based on the 6 terms related to androgen, 75 androgen-related genes were identified, among which, 16 were significantly differentially expressed (DE) between tumor and control groups (adjusted *P* < 0.05, Fig. 1A). Following that, a regulatory network of m6A and DE androgen-related genes was established. As shown in Fig. 1B, YTHDF1 was included in this network, and regulated three androgen-related genes (TRIM68, HEYL and TIPARP); thus, it was selected for the following experiments.

Furthermore, the effects of AR inhibitor on PC progression were investigated. It was found that the YTHDF1 expression was significantly up-regulated in the PC cell lines (LNCAP, DU145, and PC-3) compared to the RWPE-2 cells (*P* < 0.05), as well as in the LNCAP cells; the YTHDF1 expression was the highest (Fig. 1C). Therefore, LNCAP cells were chosen for subsequent experiments. After that, different concentrations of AR inhibitor were used to treat the LNCAP cells, and cultured for 48 h. After incubation, we found that the viability of LNCAP cells was significantly decreased when the cells treated with 5, 10, 50, and 100 μmol/L AR inhibitor in comparison with the blank cells (*P* < 0.05); and 10 μmol/L of AR inhibitor was chosen as the optimal concentration (Fig. 1D).

After that, the mRNA expression of the m6A-related genes (*METTL3*, *METTL14*, *WTAP*, *FTO*, *ALKBH5*, *YTHDF1* and *YTHDC1*) associated with AR in PC was determined by quantitative real-time PCR (qRT-PCR). After the addition of AR inhibitor, the mRNA levels of *AR*, *METTL3*, *METTL14* and *YTHDF1* were significantly down-regulated, while the expression level of *FTO* was significantly up-regulated compared to the control cells (*P* < 0.05, Fig. 1E). Western blot showed that the protein expression levels of AR and YTHDF1 were both significantly down-regulated in the AR inhibitor treated group (Fig. 1F).

### *YTHDF1 knockdown decreased AR level, viability and m6A methylation level of PC cells *in vitro

**Fig. 2:**
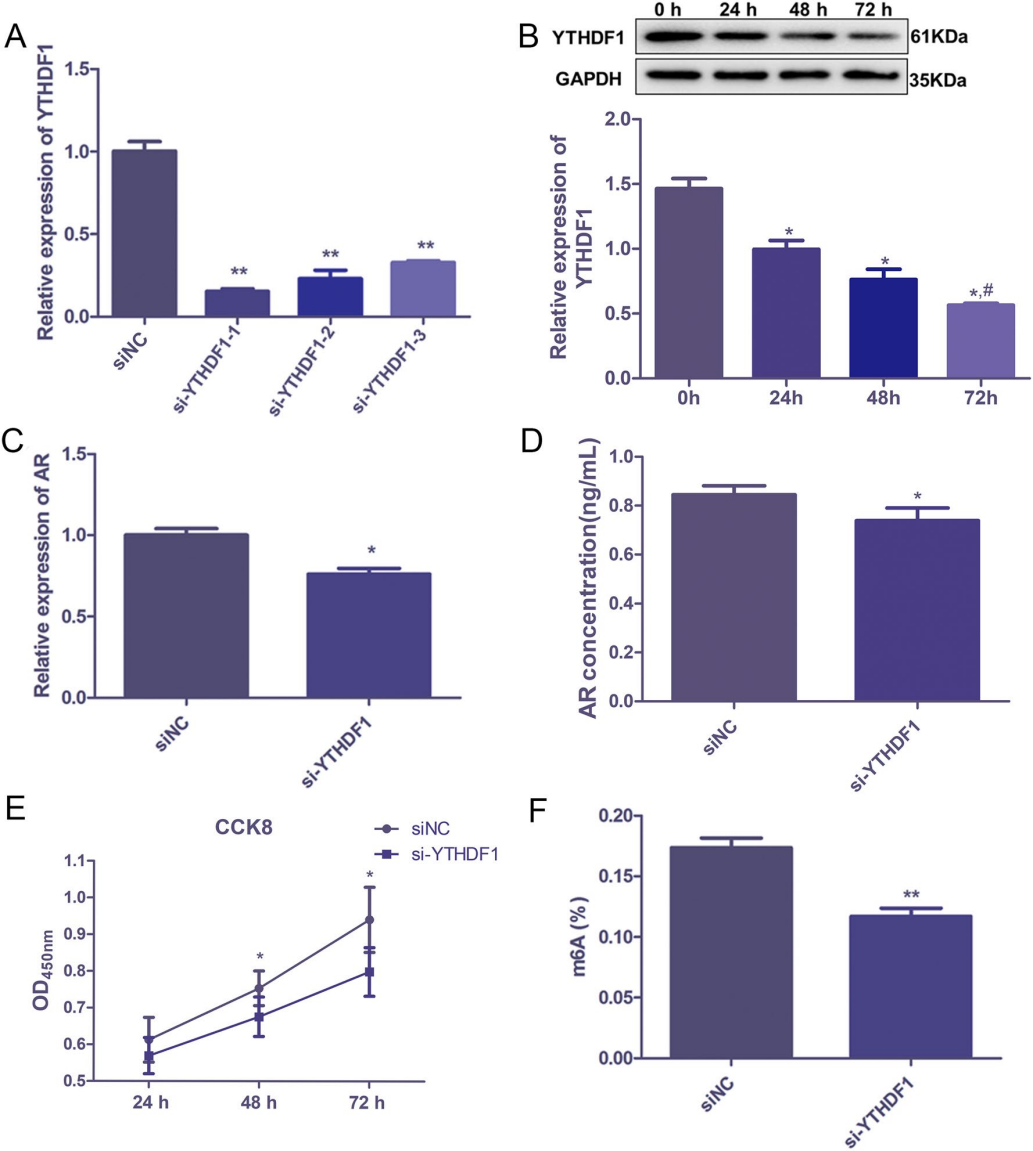
YTHDF1 knockdown decreased the AR level, viability and m6A methylation level of LNCAP cells in vitro. **A** mRNA expression of YTHDF1 in LNCAP cells after transfected with si-YTHDF1-1/2/3. ** *P* < 0.01, compared to siNC group. **B** Protein expression of YTHDF1 after transfected with si-YTHDF1-1 after cultured for 24 h, 48 h, and 72 h. *P < 0.05, compared to 0 h; ^#^*P* < 0.05, compared to 24 h. group. **C** AR mRN expression in LNCAP cells after YTHDF1 knockdown. **D** AR concentration in LNCAP cells after YTHDF1 knockdown by enzyme-linked immunosorbent assay. **E** Viability of LNCAP cells after YTHDF1 knockdown with different culture time detected by cell counting kit-8. **F** m6A methylation level after YTHDF1 knockdown detected by a RNA Methylation Quantification Kit. *P < 0.05, ** *P* < 0.01, compared to siNC group

The functions of YTHDF1 on AR in PC were further explored by suppressing its expression through siRNA targeting YTHDF1 (si-YTHDF1). After transfection, the *YTHDF1* mRNA expression was significantly lower than that in the siNC group (*P* < 0.05), and was lowest in the si-YTHDF1-1-transfected cells compared to the si-YTHDF1-2 and si-YTHDF1-3 (Fig. 2A), so si-YTHDF1-1 was selected for following study. Then, we determined the YTHDF1 protein expression in the transfected cells at different culture times, and found that within 72 h culture time, the YTHDF1 protein expression was significantly down-regulated (Fig. 2B). These indicated that the LNCAP cells with YTHDF1 knockdown were successfully established (Fig. 2B). Furthermore, *AR* expression and AR concentration in LNCAP cells after YTHDF1 knockdown were determined, and it was obvious that YTHDF1 knockdown evidently decreased the *AR* mRNA expression and AR concentration in LNCAP cells (*P* < 0.05, Fig. 2C, D). In addition, the viability of LNCAP cells was increased with the increasing culture time, as well as compared to the siNC group, and the viability of LNCAP was evidently declined in response to YTHDF1 knockdown (*P* < 0.05, Fig. 2E). Finally, we found that the m6A methylation level was also significantly declined in the si-YTHDF1-transfected LNCAP cells in comparison with the siNC cells (*P* < 0.01, Fig. 2F). These results indicated that YTHDF1 knockdown could decrease the AR level, viability and m6A methylation level of LNCAP cells.

### m6A-sequencing identified TRIM68 as a candidate target of YTHDF1

**Fig. 3:**
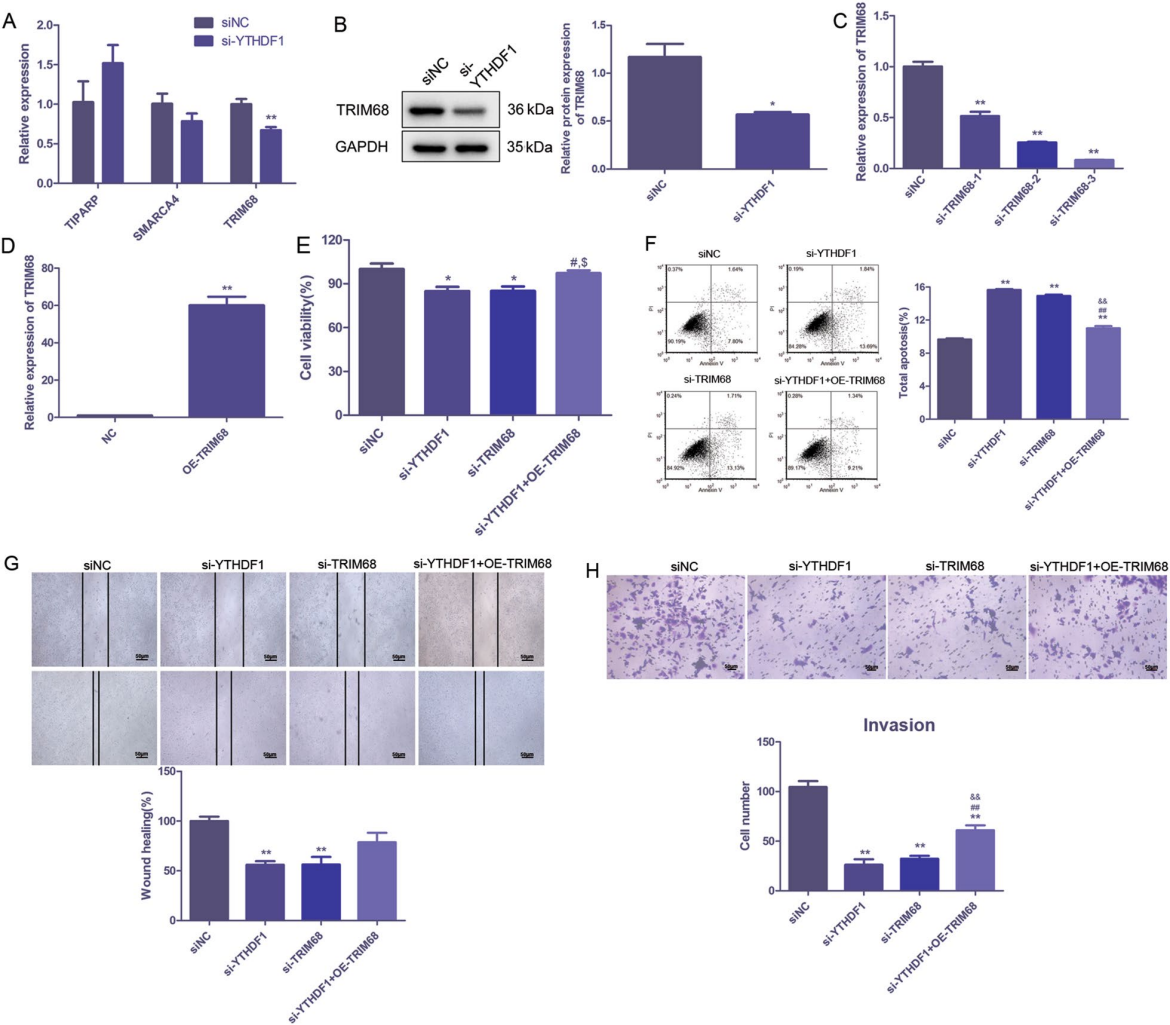
m6A-sequencing identified TRIM68 as a direct target of YTHDF1. **A** Expression levels of TIPARP, TRIM68 and SMARCA4 after YTHDF1 knockdown were detected by qRT-PCR. **B**. Expression level of TRIM68 after YTHDF1 knockdown was detected by western blot. **A**, **B** Expression of TRIM68 after transfected with siRNA and TRIM68-overexpressed plasmid was detected. Cell viability (**E**), apoptosis (**F**), wound healing (**G**), and invasion (**H**) in si-YTHDF1, si-TRIM68 and si-YTHDF1 + TRIM68-overexpression (OE) groups were detected by CCK-8, flow cytometry, wound healing and transwell assays, respectively. Scale bar = 50 μm. *P < 0.05, ** *P* < 0.01, compared to siNC group;^#^*P* < 0.05, ^##^ *P* < 0.01, compared to YTHDF1 siRNA group; ^&^*P* < 0.05, ^&&^ *P* < 0.01, compared to TRIM68 siRNA group

To investigate the molecular mechanism of YTHDF1 affecting AR in PC, we conducted m6A RNA immunoprecipitation (MeRIP) sequencing and bioinformatics analysis. MeRIP sequencing identified 21,885 m6A methylation genes in si-YTHDF1 group (Additional file 1: Table S1). Three genes (SMARCA4, TIPARP and TRIM68) were overlapped between the MeRIP sequencing data and androgen-related genes in the regulatory network above. Thus, SMARCA4, TIPARP and TRIM68 were selected for the following verification. qRT-PCR was applied to detect the expression of the three genes following YTHDF1 knockdown in LNCAP cells. The results showed that TRIM68 was significantly down-regulated after YTHDF1 knockdown (*P* < 0.01, Fig. 3A). Western blot further verified its downregulation by si-YTHDF1 (*P* < 0.05, Fig. 3B). Taken together, TRIM68 was selected as a candidate target of YTHDF1 mediated m6A modification.

### TRIM68 served as an oncogene and reversed the effects of YTHDF1 knockdown on PC in vitro

To further investigate the function of TRIM68 in PC, the expression of TRIM68 in LNCAP cells was down-regulated or up-regulated using siRNA targeting TRIM68 (si-TRIM68) or TRIM68-overexpressed plasmid (OE-TRIM68) (Fig. 3C, D). CCK-8 assay revealed that both si-YTHDF1 and si-TRIM68 reduced the PC cell viability (*P* < 0.05), while after overexpression of TRIM68 on the basis of YTHDF1 knockdown, the cell activity was increased (*P* < 0.05, Fig. 3E). Results of flow cytometry assay showed that after YTHDF1 or TRIM68 knockdown, the apoptosis of LNCAP cells increased significantly (*P* < 0.01). After overexpression of TRIM68 on the basis of YTHDF1 knockdown, the apoptosis of PC cells was significantly reduced compared with si-YTHDF1 and si-TRIM68 groups (*P* < 0.01, Fig. 3F). Wound healing assay revealed that YTHDF1 or TRIM68 knockdown significantly decreased cell migration ability (*P* < 0.01), while TRIM68 overexpression reversed the effects of YTHDF1 knockdown on migration of LNCAP cells (Fig. 3G). Transwell assay identified similar results (*P* < 0.01, Fig. 3H).

**Fig. 4:**
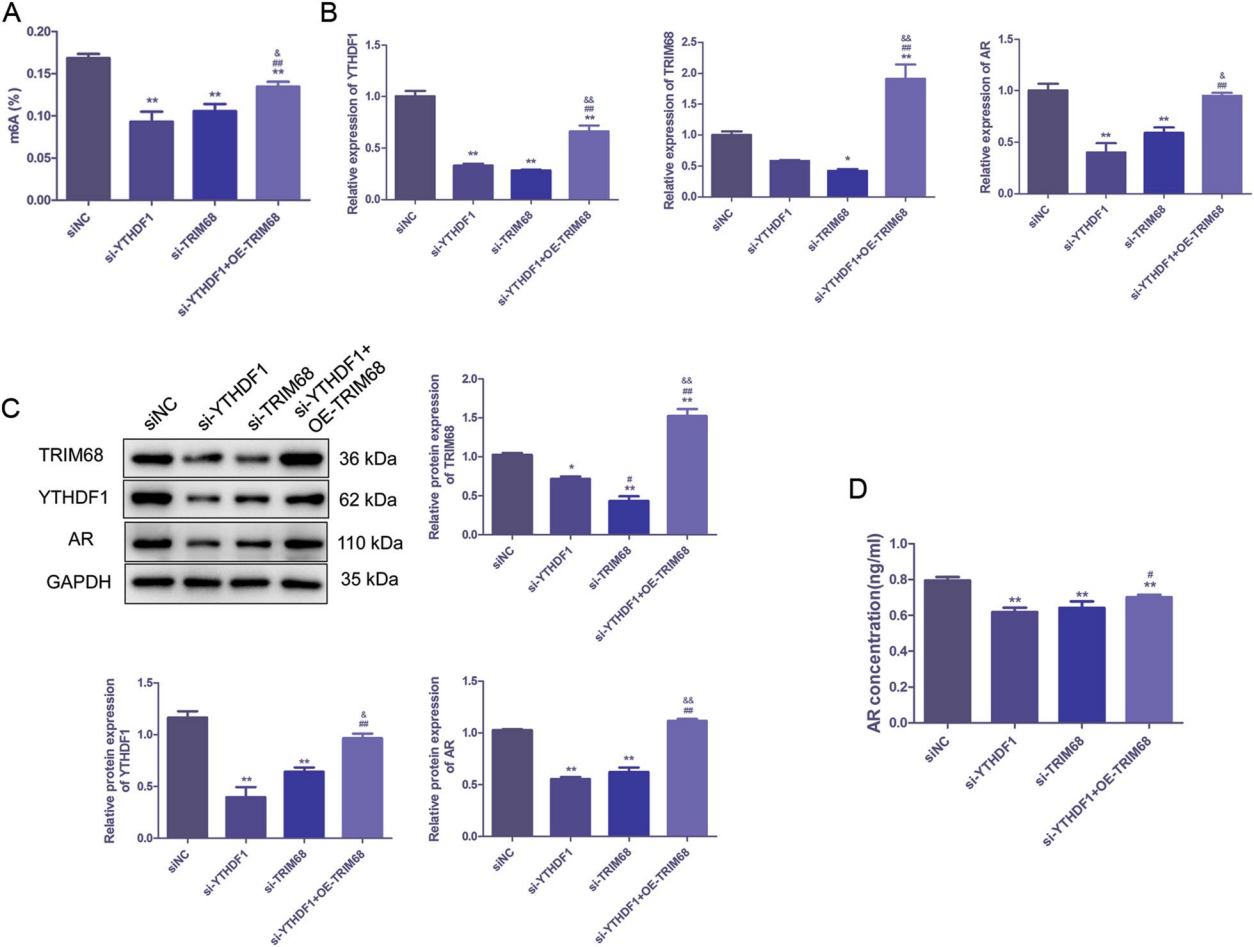
TRIM68 reversed the effects of YTHDF1 knockdown in PC in vitro. **A** m6A methylation level in PC cells of si-YTHDF1, si-TRIM68 and si-TRIM68 + OE-TRIM68 groups. **B**, **C** mRNA and protein levels of YTHDF1, TRIM68 and AR in PC cells of si-YTHDF1, si-TRIM68 and si-TRIM68 + OE-TRIM68 were detected by qRT-PCR and western blot. **D** Level of AR in cell supernatant in si-YTHDF1, si-TRIM68 and si-TRIM68 + OE-TRIM68 groups was detected by ELISA. *P < 0.05, **P < 0.01, compared to siNC group; ^#^*P* < 0.05, ^##^*P* < 0.01, compared to YTHDF1 siRNA group; ^&^*P* < 0.05, ^&&^*P* < 0.01, compared to TRIM68 siRNA group

Furthermore, the effects of TRIM68 on the m6A methylation level and AR level of PC cells in vitro were also investigated. As presented in Fig. 4A, C, knockdown of YTHDF1 or TRIM68 significantly decreased the m6A methylation level, and the mRNA and protein levels of YTHDF1, TRIM68 and AR in LNCAP cells (*P* < 0.01), while TRIM68 overexpression reversed the effects of YTHDF1 knockdown on LNCAP cells (*P* < 0.01). In addition, overexpression of TRIM68 could also reverse the effect of YTHDF1 knockdown on the level of AR in cell supernatant (*P* < 0.01, Fig. 4D).

### TRIM68 reversed the effects of YTHDF1 knockdown in PC in vivo

**Fig. 5:**
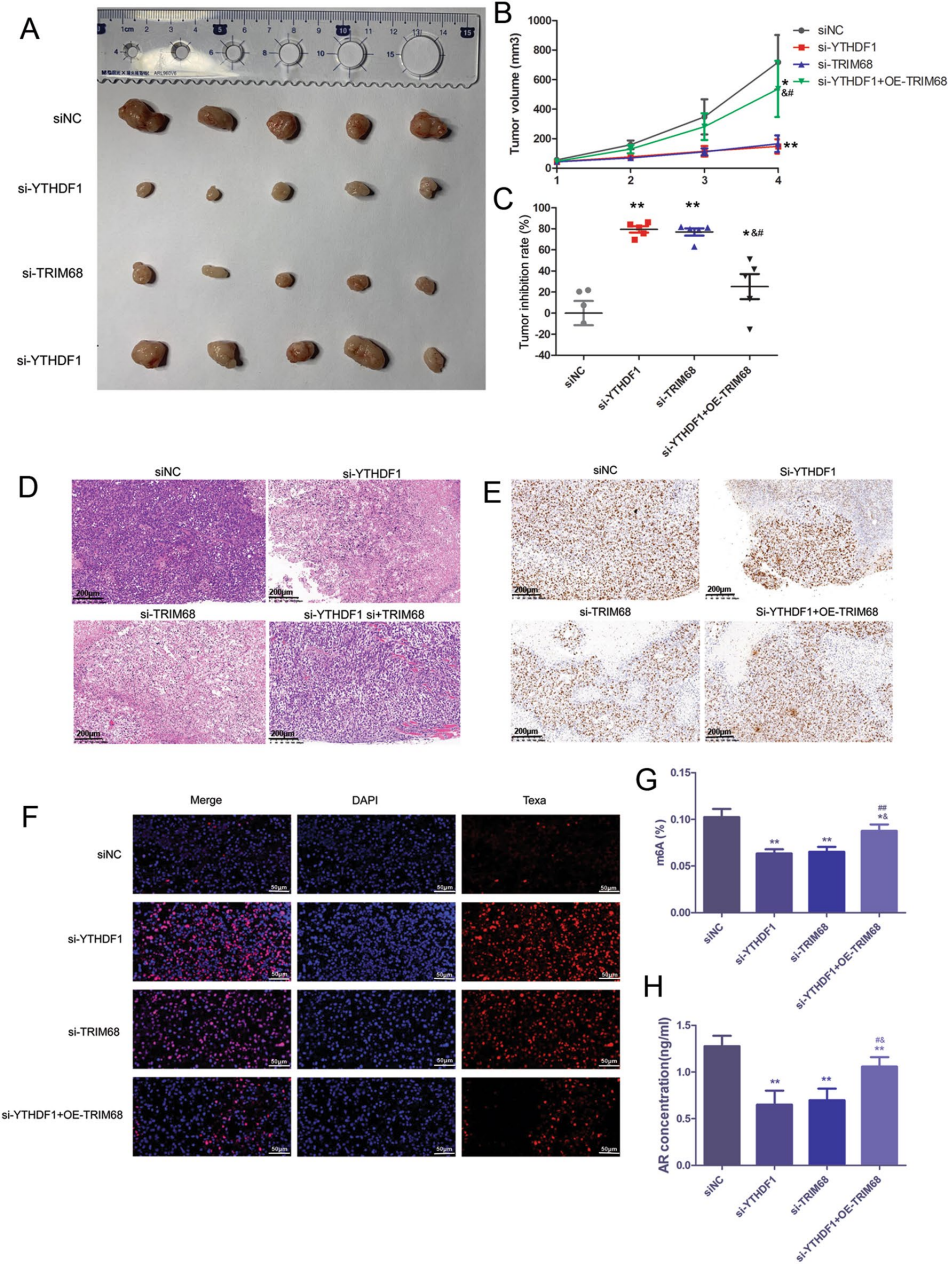
TRIM68 reversed the effects of YTHDF1 knockdown in PC in vivo. Representative photos of tumors (**A**), tumor volume (**B**), tumor inhibition rate (**C**), H&E (**D**), immunohistochemistry (**E**) and TUNEL staining (**F**) in si-YTHDF1, si-TRIM68 and si-TRIM68 + OE-TRIM68 groups. Scale bar for H&E and immunohistochemistry was 200 μm; and scale bar for TUNEL staining was 50 μm. The m6A (**G**) and AR level (**H**) in nude mouse xenografts and serum in si-YTHDF1, si-TRIM68 and si-TRIM68 + OE-TRIM68 groups. *P < 0.05, **P < 0.01, compared to siNC group;^#^*P* < 0.05, ^##^*P* < 0.01, compared to YTHDF1 siRNA group; ^&^*P* < 0.05, ^&&^*P* < 0.01, compared to TRIM68 siRNA group

Finally, we studied the effect of YTHDF1 and TRIM68 on PC in vivo. The results showed that the tumor volume decreased significantly after YTHDF1 or TRIM68 knockdown, while overexpression of TRIM68 on the basis of YTHDF1 knockdown could promote tumor growth (Fig. 5A–C). Compared with the siNC group, the number of inflammatory cells significantly decreased and of normal cells significantly increased in the si-YTHDF1 or si-TRIM68 groups, while in YTHDF1 knockdown + TRIM68 overexpression group, the number of inflammatory cells increased compared with that in the si-YTHDF1 or si-TRIM68 groups (Fig. 5D). IHC revealed that the Ki67-positive staining cells in the prostate tissue of si-YTHDF1 and si-TRIM68 groups were significantly reduced (*P* < 0.05), and in YTHDF1 knockdown + TRIM68 overexpression group were reduced not significantly compared with those in siNC group (Fig. 5E). TUNEL results suggested that there were more apoptotic cells in the si-YTHDF1 group, and si-TRIM68 group, compared with those in siNC group. In the si-YTHDF1 + OE-TRIM68 group, the number of apoptotic cells was significantly reduced compared with that in the si-YTHDF1 group and si-TRIM68 group (*P* < 0.05), but still more than that in the siNC group (Fig. 5F). For the AR level in nude mouse serum, YTHDF1 or TRIM68 knockdown decreased the AR level while of TRIM68 overexpression could reverse the effect of YTHDF1 knockdown in vivo (*P* < 0.05, Fig. 5G). In addition, YTHDF1 or TRIM68 knockdown significantly decreased the m6A methylation level in xenografts (*P* < 0.01). After overexpression of TRIM68 on the basis of YTHDF1 knockdown, the m6A methylation level in xenografts of nude mice was increased (*P* < 0.05, Fig. 5H).

## Discussion

As the most common chemical methylation on mRNAs in human, m6A regulators, including writers, easers and readers, have recently been considered essential for biological regulation of human cancers [9, 15]. PC is one of the leading malignant tumors among male population [16, 17]. Our study for the first time uncovered a direct role of m6A-modified-binding protein YTHDF1 in regulating androgen function-related genes by regulating TRIM68 in PC.

As we known, PC is a hormonally regulated malignancy, and AR plays a key role in the progression of PC [18]. AR is a transcription factor and can collaboratively regulate the effects of androgens with some co-regulatory factors. AR up-regulates or down-regulates the expression of target genes through coactivators or co-repressors [19, 20]. The activity of AR and coregulatory factors is regulated by post-translational modifications such as methylation, and ubiquitination [21, 22]. Interestingly, the methylation modification has been proved in our study. Previous studies have found that expression of METTL3 and WTAP are regulated by androgen in PC cell lines, as well as increased expression of METTL3 plays a pro-tumor role in PC, while METTL3 silencing resulted in upregulation of AR, together with 134 AR-regulated genes [23, 24]. Another study showed that the overall level of m6A in castration resistance PC was increased compared to the castration sensitive PC, and METTL3 could activate ERK pathway, and induce the resistance to AR inhibitor (Enzalutamide) in PC. Taken together, it can be inferred that alteration of m6A levels is closely connected with the occurrence and development of PC.

It has been reported that the translation of AR mRNA is coordinated and regulated by RNA-binding proteins YTHDF3 and G3BP1; as well as m6A-modified AR mRNA is bound to YTHDF3 and translationally stimulated, whereas m6A-unmodified AR mRNA is bound with G3BP1 and translationally inhibited [25]. Another study manifested that YTHDF1, YTHDC2, and YTHDF2 were up-regulated in PC, and had positive correlation with the Gleason grades of PC, as well as the m6A levels were higher in the LNCAP cells [26] Furthermore, m6A regulators are reported to have positive correlation with AR, and play important roles in PC progression [26]. YTHDF1, similar with YTHDF3, is also the most abundant reader of m6A-modified mRNA, which functionally connects m6A-modified mRNA to its fate [27]. YTHDF1 is necessary for protein translation [28–30]. Many studies have reported that YTHDF1 plays a critical role in tumor biology by influencing the expression of some key factors or by regulating the protein translation of key genes associated with the important signaling pathways[31–33]. YTHDF1 has been demonstrated to promote the metastasis of gastric cancer in an m6A-dependent way by promoting USP14 translation [34]. Wang et al. [35] have revealed that YTHDF1 can aggravate the progression of cervical cancer by m6A-mediated up-regulation of RANBP2. Liu et al. [36] reported that YTHDF1 promotes ovarian cancer progression via augmenting EIF3C translation. Recently, Li et al. [37] demonstrated that YTHDF1 was overexpressed in PC cells, knockdown of which suppressed the proliferation, and invasion of PC cells by regulating TRIM44. Another study also reported that YTHDF1 was up-regulated in PC tissue and was related to an adverse prognosis in patients with PC [38]. However, these studies focused on the function of YTHDF1 in cancer progression, its role in androgen function has not been reported to our knowledge. Our results revealed that YTHDF1 was down-regulated in AR inhibitor groups. Knockdown of YTHDF1 suppressed the AR level, cell viability, migration, and invasion, and increased the apoptosis of PC cells. In vivo experiments obtained similar results. These results, together with the reports, we speculate that YTHDF1 may function as a carcinogenic factor by increased the AR level in PC, and co-action of AR signaling system and m6A modification may play essential roles in the development and progression of PC.

To further reveal the possible molecular mechanisms of YTHDF1 affecting AR in PC, we conducted MeRIP sequencing and bioinformatics analysis. Based on our results, TRIM68 was selected as a candidate target of YTHDF1. TRIM68 belongs to the tripartite motif-containing protein family that is defined by the common domain structure of a B-box, a Ring finger, and a coiled-coil motif [39]. A previous study has demonstrated that TRIM68 is preferentially expressed in PC cells. It acts as a coactivator of AR through its ubiquitin ligase activity. Overexpression of TRIM68 could enhance AR-mediated transcriptional activation, while knockdown of TRIM68 could inhibit AR-mediated transcriptional activation in PC cells [40]. In this study, we revealed that knockdown of TRIM68 alone had similar role with YTHDF1 knockdown in PC cells. Importantly, TRIM68 overexpression could reverse the effects of YTHDF1 knockdown in PC both in vitro and in vivo. To our best knowledge, our study for the first time demonstrated that relationship between TRIM68 and m6A methylation (YTHDF1) in PC.

However, there are some limitations in our study. First, the roles of YTHDF1 or TRIM68 in PC should be further investigated in the androgen adding system. Second, the reasons for the decreased m6A modification after YTHDF1 knockdown (whether modify the expression of m6A writers or erasers) remain unclear, and need to be explored in the future. In addition, the further in-depth mechanism research of YTHDF1 and TRIM68 (including mRNA transcription, translation, stability, and their upstream and downstream relationships) is also necessary to be unearthed in the future through various techniques, such as immunofluorescence staining localization, RIP-qPCR, and rescue assays.

In conclusion, our study demonstrated the key role of YTHDF1-mediated m6A modification in PC progression by regulating androgen function-related gene TRIM68 in PC. The findings will aid in the development of therapeutic strategies against PC.

## Materials and methods

### Cell culture

Human prostate normal cells (RWPE2), and PC cell lines LNCAP, DU145 and PC-3 cells were purchased from Cell Bank of the Chinese Academy of Sciences (Shanghai, China). The RWPE2 cells were maintained in the defined keratinocyte–SFM (Thermo Fisher Scientific, USA) containing with 10% fetal bovine serum (FBS, Thermo Fisher Scientific) and 1% penicillin/streptomycin (Thermo Fisher Scientific); as well as the LNCAP, DU145 and PC-3 cells were maintained in RPMI-1640 medium (Thermo Fisher Scientific) supplemented with 10% FBS and 1% penicillin/streptomycin. All the cells were cultured in an incubator with 5% CO_2_ at 37 °C.

### Cell counting kit-8 (CCK-8) assay

Cells were seeded in a 96-well plate (1 × 10^4^ cells/well) overnight. The cells were then treated with different concentrations of AR inhibitor (ARN-509; SD0033-10 mM, Beyotime, China) (0, 1, 5, 10, 50, 100 μM) for 48 h. After that cells were treated with 10 µl of CCK-8 reagent (C0038; Beyotime, China) for 2 h. The absorption values were measured at 450 nm. The cell viability curve was plotted.

### Quantitative real-time PCR (qRT-PCR)

For cells, total RNA was isolated from the cells with different treatments (n = 3 for each group) using RNAiso Plus (Takara, Japan) following the manufacturer’s instructions. Then, the isolated total RNA was reverse transcribed into cDNA with PrimeScript RT Master Mix (Takara). The temperature protocol of reserve transcription was 37 °C for 60 min and 85 °C for 5 s. After that, Power SYBR Green PCR Master Mix (Thermo, Waltham, MA, USA) was used to perform a real-time PCR according to the manufacturer’s protocols. The reaction was initiated at 50 °C for 2 min, a total of 40 cycles at 95 °C for 2 min, 95 °C for 15 s, and at 60 °C for 60 s, and then melted at 95 °C for 15 s, 60 °C for 60 s, and 95 °C for 15 s. The primers used in this experiment are shown in Table 1, and GAPDH was used as the reference gene. The relative changes in the transcriptional levels of all genes were calculated using 2^−∆∆Ct^ method.

**Table 1:** Primers used in this study

Gene	Direction	Sequencing (5’–3’)
AR	Forward	CCAGGGACCATGTTTTGCC
AR	Reverse	CGAAGACGACAAGATGGACAA
METTL3	Forward	TTGTCTCCAACCTTCCGTAGT
METTL3	Reverse	CCAGATCAGAGAGGTGGTGTAG
METTL14	Forward	AGTGCCGACAGCATTGGTG
METTL14	Reverse	GGAGCAGAGGTATCATAGGAAGC
WTAP	Forward	CTTCCCAAGAAGGTTCGATTGA
WTAP	Reverse	TCAGACTCTCTTAGGCCAGTTAC
FTO	Forward	ACTTGGCTCCCTTATCTGACC
FTO	Reverse	TGTGCAGTGTGAGAAAGGCTT
ALKBH5	Forward	CGGCGAAGGCTACACTTACG
ALKBH5	Reverse	CCACCAGCTTTTGGATCACCA
YTHDF1	Forward	ACCTGTCCAGCTATTACCCG
YTHDF1	Reverse	TGGTGAGGTATGGAATCGGAG
YTHDC1	Forward	AACTGGTTTCTAAGCCACTGAGC
YTHDC1	Reverse	TGTGCAGTGTGAGAAAGGCTT
SMARCA4	Forward	GACCAGCACTCCCAAGGTTAC
SMARCA4	Reverse	CTGGCCCGGAAGACATCTG
TIPARP	Forward	AGAACGAGTGGTTCCAATCCA
TIPARP	Reverse	TGGGTGCAAAAGATCAGTCTG
TRIM68	Forward	GGAGCCCATGAGCATTGACT
TRIM68	Reverse	GACAGGTGTAACCCCAGTTCT
GAPDH	Forward	TGACAACTTTGGTATCGTGGAAGG
GAPDH	Reverse	AGGCAGGGATGATGTTCTGGAGAG

### Western blot

Cells with different treatments (n = 3 for each group) were homogenized with RIPA solution (Beyotime) on the ice for 30 min. After centrifugation at 12,000 g for 10 min, the total protein concentrations were measured by a BCA Protein assay kit (Beyotime). Then, the protein samples (20 μg) were separated by 10% SDS–PAGE, and transferred to the PVDF membranes (IPVH00010; Millipore, USA). After being blocked in 5% skim milk at 37 °C for 1 h, the membranes were incubated with the primary antibodies (anti-AR (1:5000; 22,089-1-AP; Proteintech, China), anti-YTHDF1 (1:1000; 17,479–1-AP; Proteintech), and TRIM68 (1:1000; bs-17123R; Bioss, Warsaw, Poland)) at 4 ℃ overnight, and then incubated with the secondary antibody (1:5000; 111–035-003; Jackson immune research, USA) at 37 °C for 2 h. Finally, the expression levels of the related proteins were detected by an ECL kit, and visualized by Image Quant LAS 4000mini (GE Healthcare, USA).

### Plasmid preparation

LB agar plates were prepared using 3.2 g LB Broth Agar and 80 mL ddH_2_O, followed by autoclave-based sterilization. Then, the media were cooled to below 60 °C, and ampicillin was added to a final concentration of 100 μg/mL. The LB agar media was prepared using 7.5 g LB Broth Medium and 300 mL ddH_2_O, followed by autoclave-based sterilization.

Under sterile conditions, 1 μL of plasmids (50–100 ng) were added into DH5α competent cells and placed on ice for 30 min. After standing at 42 °C for 90 s, cells were quickly transferred to the ice for 3–5 min. Then, 800 μL LB agar media (without ampicillin) was added and shaken at 100–150 rpm for 50 min. After that, 200 μL liquid was sucked out and added into solid LB agar plate (containing ampicillin), and cultured in 37 °C incubator overnight for 14–18 h. After transformation, monoclonal colonies were selected with sterile toothpick into liquid LB medium containing ampicillin and shaken gently for 8 h (220 rpm) at 37 °C. Plasmid DNA was extracted using the Endo-Free Plasmid Midi kit (Omega Bio-Tek, Norcross, GA, USA).

### Cell transfection

The cells were re-suspended with complete culture medium, and then seeded into 24-well plates at 4 × 10^4^ cells/well. The plasmids above were co-transfected using the Lipofectamine 2000 reagent (11,668–027; Thermo) in accordance with the instruction of the manufacturer.

### Flow cytometry

The cells of each group were digested with trypsin. After centrifugation, cells were collected and apoptosis rates were detected by flow cytometry using an Annexin V-FITC/PI detection kit (556,420; BD Biosciences, Boston, MA, USA).

### Wound healing assay

Horizontal lines (about 1 cm) were drawn on the back of the 6-well plate with marker. The cells were inoculated in the well. On the second day, the sterile pipette tip was utilized to scratch (perpendicular to the horizontal line on the back). The medium was removed, and washed with phosphate buffer saline (PBS), and then serum-free medium was added for incubating. Photographs were taken at 0 and 48 h, respectively.

### Transwell assay

The transfected cells were digested with trypsin, and then centrifugated at 1000 rpm for 5 min. The supernatant was removed and resuspended with 5 mL sterile PBS. A small number of cells were taken and counted with a blood cell counting plate. The cells were centrifuged at 1000 rpm for 5 min and 3 mL serum-free medium was added to re-suspend the cells, and cell density was adjusted to 2 × 10^5^/ml. Then 500 μL complete medium was added into the 24-well plates. The porous membrane was coated with Matrigel and 200 μL cells suspension were seeded into the upper chamber. After 48 h, the cells on the upper surface of the filter were removed, and that invaded to the lower surface were fixed and stained with 0.5% crystal violet, and counted under a light microscope.

### Analysis of androgen-related genes in PC

PC transcriptome expression level data were obtained from TCGA database. The test platform was Illumina HiSeq 2000 RNA Sequencing. There were 551 samples in the dataset, and 548 (496 tumor and 52 normal) samples were included in the analysis after corresponding to the clinical information of PC samples. In addition, 6 androgen-related function terms (“androgen catabolic process”, “androgen biosynthetic process”, “androgen metabolic process”, “androgen receptor signaling pathway”, “regulation of androgen receptor signaling pathway”, and “negative regulation of androgen receptor signaling pathway”) were downloaded from the MSigDB module in Gene Set Enrichment Analysis (GSEA) database. The genes in these terms were considered as androgen-related genes.

Then, according to the sample information, the differentially expressed (DE) androgen-related genes between tumor and control samples were selected using R3.6.1 limma 3.34.7. The targets of m6A were downloaded from m6A2Target database, and the significantly DE androgen-related genes were mapped to the target genes to construct a regulatory network.

### m6A RNA immunoprecipitation (MeRIP) sequencing

RNAs were extracted as above. The mRNA was fragmented to approximately 200 nt using RNA fragment reagent. A 5 μg fragment of mRNA was stored as input control of RNA sequence, and a 50 μg fragment of mRNA was used for MeRIP. Both the m6A IP samples and the input samples without IP were used to generate libraries for RNA sequencing. Libraries were sequenced on an Illumina Novaseq^™^ 6000 platform (LC-Bio Technology CO., Ltd., Hangzhou, China) with paired-end reads.

Sequence quality of IP and input samples were analyzed using FastQC and RseQC. HISAT2 was used for reference genome mapping. StringTie was used to analyze the expression level for all transcripts and genes from input libraries by calculating FPKM.

### RNA m6A quantification

The m6A modification was detected using the EpiQuik m6A RNA Methylation Quantification Kit (P-9005; Epigentek Group Inc., USA). Briefly, 2 µL of negative control and 200 ng of RNA were added into strip wells. m6A was detected by capture and detection antibodies. The detected signal was enhanced and colorimetrically quantified at 450 nm.

### Enzyme-linked immunosorbent assay (ELISA)

The AR level in supernatant was determined using the AR ELISA kit (ELK1829-1; ELK Biotechnology, Wuhan, China). The AR concentration was detected at a wavelength of 450 nm.

### Subcutaneous xenografts of nude mice

Cells (si-NC group, si-YTHDF1 group, si-TRIM68 group, si-YTHDF1 + OE-TRIM68 group) were digested with 0.25% trypsin, and fetal bovine serum was added to prevent excessive digestion. After centrifugation at 1000 rpm for 5 min, cells were collected and suspended with culture medium without serum, and cell density was adjusted to 5 × 10^7^ cells/ml.

SPF male BALB/c-nu nude mice (*n* = 20) were used in this study. Tumor cells (100 μl) were inoculated subcutaneously in the axils of the right forelimbs of mice. After the injection, the mental state, activity, diet, urine and feces of the nude mice were observed regularly every day. The body weight was measured weekly with an electronic balance, and the diameters of subcutaneous graft were measured weekly with vernier caliper. After the tumor grew to about 40 mm^3^, the nude mice were given flutamine (100 mg/kg; continuous administration for 1 week). The subcutaneous graft tumor volume V (mm^3^) = longest tumor diameter (mm) × shortest tumor diameter (mm) × 0.5. According to the tumor volume obtained, the growth curve of transplanted tumor was plotted.

Five weeks later, the mice were weighed, and sacrificed by cervical dislocation method. The tumor was separated and rinsed with sterile PBS, and then weighed. After weighing, whole blood was taken to separate serum, and tumor tissue was taken. One part was fixed in 4% paraformaldehyde solution, and the other part was stored at -80 °C.

The animal experiments were conducted according to the National Institutes of Health Guide for the Care and Use of Laboratory Animals and approved by Institutional Animal Care and Use Committee of Ganzhou people's Hospital.

### Hematoxylin and eosin (H&E) staining and immunohistochemistry (IHC)

Prostate tissues were fixed in 4% paraformaldehyde, followed by rinsing with distilled water. The fixed tissues were embedded in paraffin, and sectioned (thickness: 4 μm). Then, sections were dewaxed and rehydrated, and then stained with H*&*E (Sigma, USA).

For IHC, the sections were blocked in 5% bovine serum albumin and then incubated with Ki67 antibodies (Cell signaling technology, MA, USA; Cat. #9027; 1:100) at 4 °C overnight, followed by incubation with secondary antibody for 80 min at 25 °C. Following that, 3,3′-diaminobenzidine development (DAB) and hematoxylin staining were carried out. The images were obtained with Olympus IX71 inverted microscope (Olympus, Japan).

### Terminal deoxynucleotidyl transferase dUTP nick end labelling (TUNEL) assay

TUNEL staining was performed using In situ cell death detection kit (11,684,795,910, Roche, Switzerland). Briefly, prostate tissue sections were permeabilized with proteinase K labeled with terminal deoxynucleotidyl transferase containing digoxigenin–dUTP and incubated with 3% hydrogen peroxide solution. Nuclear were stained with DAPI, and the positive cells were observed using immunofluorescence microscope (Nikon Eclipse 50i, Japan).

### Statistical analyses

Data are presented as means ± standard deviation. Student *t* test was used for the comparison of two groups and one-way ANOVA for three or more groups. These analyses were based on Graphpad prism 5 (Graphpad Software, San Diego, CA, USA). *P* < 0.05 was considered significant.

### Supplementary Information

**Additional file 1: Table S1.** List of 21,885 m6A methylation genes in si-YTHDF1 group detected by MeRIP sequencing.

## Data Availability

The datasets generated and/or analysed during the current study are available in the manuscript and its supplementary files.

## Abbreviations

AR: Androgen receptor
PC: Prostate cancer
MeRIP: M6A RNA immunoprecipitation
m6A: N6-methylladenosine
CCK-8: Cell counting kit-8
DE: Differentially expressed
ELISA: Enzyme-linked immunosorbent assay
H&E: Hematoxylin and eosin
IHC: Immunohistochemistry
TUNEL: Terminal deoxynucleotidyl transferase dUTP nick end labelling
